# Riboswitch theo/*metE* as a Transcription Regulation Tool for *Xanthomonas citri* subsp. *citri*

**DOI:** 10.3390/microorganisms9020329

**Published:** 2021-02-06

**Authors:** Danilo Bueno, Danielle B. Pedrolli, Paula M. M. Martins, Daniela A. Bocchini, Karen C. M. Moraes, Agda P. Facincani, Jesus A. Ferro, Alessandro M. Varani, Michelle M. Pena, Henrique Ferreira

**Affiliations:** 1 Department of General and Applied Biology, Biosciences Institute, São Paulo State University (UNESP), Rio Claro, SP 13506-900, Brazil; danilo_riber@yahoo.com.br (D.B.); kcm.moraes@unesp.br (K.C.M.M.); 2 Department of Biological Sciences, School of Pharmaceutical Sciences, São Paulo State University (UNESP), Araraquara, SP 14800-903, Brazil; danielle.pedrolli@unesp.br; 3 Citriculture Center, Agronomic Institute of Sao Paulo (IAC), Cordeiropolis, SP 13490-970, Brazil; pmmm@outlook.com.br; 4 Department of Biochemistry and Chemistry Technology, Chemistry Institute, São Paulo State University (UNESP), Araraquara, SP 14800-060, Brazil; daniela.bocchini@unesp.br; 5 Department of Technology, School of Agricultural and Veterinary Sciences, São Paulo State University (UNESP), Jaboticabal, SP 14884-900, Brazil; agda.facincani@unesp.br (A.P.F.); jesus.ferro@unesp.br (J.A.F.); alessandro.varani@unesp.br (A.M.V.)

**Keywords:** citrus canker, regulation of gene expression, chromosome segregation, *parB*, pNPTS138 sequence

## Abstract

*Xanthomonas citri* subsp. *citri* (*X. citri*) is the causal agent of Asiatic Citrus Canker (ACC), a disease that affects citrus. ACC has no cure, and growers must rely on special agricultural practices to prevent bacterial spreading. Understanding *X. citri* basic biology is essential to foresee potential genetic targets to control ACC. Traditionally, microbial genetics use gene deletion/disruption to investigate gene function. However, essential genes are difficult to study this way. Techniques based on small-RNAs and antisense-RNAs are powerful for gene characterization, but not yet fully explored in prokaryotes. One alternative is riboswitches, which derive from bacteria, and can control transcription/translation. Riboswitches are non-coding RNAs able to modulate gene expression in the presence of specific ligands. Here we demonstrate that the riboswitch theo/*metE* decreases *parB* expression in *X. citri* in a platform responsive to theophylline. By monitoring cell respiration, we showed that higher concentrations of the ligand interfered with bacterial viability. Therefore, we determined the safe dose of theophylline to be used with *X. citri*. Finally, in downstream investigations of *parB* transcription modulation, we show evidence for the fact that ParB is stable, remains functional throughout the cell cycle, and is inherited by the daughter cells upon cell division.

## 1. Introduction

The Gram-negative bacterium *Xanthomonas citri* subsp. *citri* [1] is the causal agent of Asiatic Citrus Canker, one of the major causes of yield losses in the production of sweet oranges in citrus growing areas around the world [2,3]. There is no treatment for citrus canker, and nowadays, control of this disease is done by the application of a set of agricultural measures intended to avoid the spread of *X. citri* amongst orchards and to areas free of its occurrence [2]. Among them, we cite the production and plantation of healthy and less susceptible citrus seedlings, the use of wind-breaks among orchards to prevent the spread of *X. citri* by the combined action of wind and rain, and the recurrent application of copper formulations to minimize bacterial infection. Moreover, the intensive study of the pathogen, and its life cycle, continues to be a good strategy that, at some point, can help the development of alternatives to control it.

The investigation of gene expression and functionality are, in general, conducted based on either transposon disruption or gene deletion/complementation seeking to understand their roles in several aspects of the cell life cycle. Although these are the basis for genetic analyses in any living organism, many drawbacks hinder effectiveness. Gene disruption is especially prone to generate polar mutations, since they are often achieved by the insertion of relatively large DNA elements into the genomes [4]. On the other hand, there are genes that cannot be deleted, since they are either essential or involved in essential traits that cannot be perturbed. A classical workaround here is the use of conditional mutants for a particular gene/protein of interest, in which cells expressing them grow normally at some cultivation conditions and lack that protein at others, non-permissive, cultivation conditions. This was how Filamentation Temperature Sensitive alleles (*fts*) were identified in *Escherichia coli*, and are now known as genes coding for cell division proteins [5]. Protein depletion is another possibility, in which essential genes are placed under the control of inducible promoters either into the chromosome of the cell-type being investigated (ectopic expression) or in a plasmid. Upon obtaining cells having this extra copy of the essential gene that will be characterized, the original one can be deleted, and modulation of gene expression from the inducible promoter allows the functional study of the essential gene. This strategy was already used in *X. citri* to characterize the protein ParB [6]. ParB organizes de bacterial centromere by binding to the ori-region of the bacterial chromosome, and it is involved in chromosome segregation in many bacteria [7]. In a few bacteria, ParB operates in conjunction with an ATPase called ParA, which forms polymers able to relocate/orient the ParB/ori-region during chromosome segregation [8,9]. Finally, *parA* and *parB* are usually organized in a small operon in bacterial chromosomes [7].

Clustered regularly interspaced short palindromic repeat (CRISPR)-based technologies, which derive from the adaptive immune systems found in bacteria and archaea, are extremely powerful and versatile tools that enable the study of gene expression not only in prokaryotes, but also in cells belonging to other kingdoms of life (reviewed by [10]). Depending on the experimental design, CRISPR effectors can bind to DNA or RNA, and consequently inhibit transcription of a gene, block transcription elongation, and lead to the elimination of mRNA molecules. According to its biological function, one of the most attractive uses CRISPR technology may have is the possibility to repress multiple targets at the same time [11]. If well refined, such property may allow broad and complex characterizations of genetic traits at once. By performing a broad genome analysis on the occurrence of CRISPR-Cas in *Xanthomonas*, Martins et al. [12] found that 60% of Xanthomonas spp. showed at least one cas operon. Jeong et al. [13] analyzed 56 isolates of *X. citri* and proposed the use of the repertoire of spacers in this plant pathogen for strain typing. Although allele exchange seems to work pretty well in *Xanthomonas* spp., CRISPR tools have the advantage of being a clean strategy, which can be used to turn gene expression ON/OFF without leaving scars around and/or within the genome sequences under investigation. As downsides, CRISPR-based tools may produce toxicity when expressed on some cells, and the possibility to interfere with genes other than the targets to which they were designed to act on [10].

**Figure 1:**
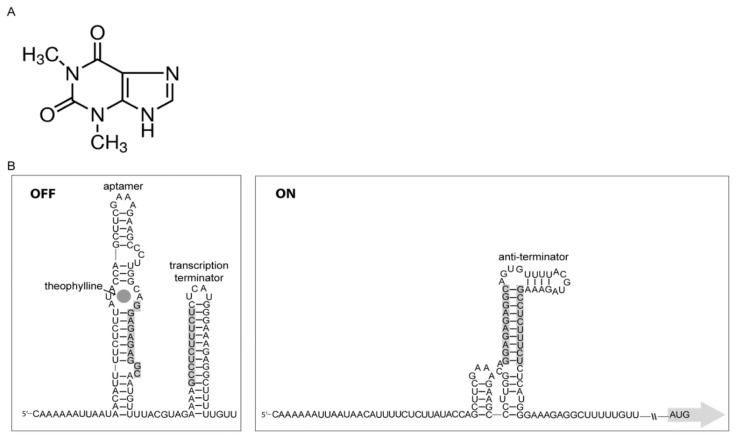
Riboswitch theo*/metE*. (**A**) The structure of theophylline, the ligand that modulates the riboswitch theo*/metE*. (**B**) Nucleotide sequence of the riboswitch theo*/metE* showing the two secondary structures adopted upon theophylline binding. In the OFF mode, the transcription terminator hairpin loop is formed by theophylline binding upstream of it, which generates the aptamer (the theophylline-binding moiety). In the ON mode, the anti-terminator structure allows the progress of the RNA polymerase.

Besides CRISPR, antisense RNA (asRNA; RNAi) and small RNA (sRNA), which are techniques that have long been explored for gene expression characterizations in eukaryotes, are also available for prokaryotes. However, there is a class of RNA elements named as riboswitches, which were discovered in bacteria with the function of controlling transcription and/or translation of diverse cellular events that include vitamin metabolism, nucleotide and amino acid biosynthesis [14,15,16]. What is unique about riboswitches is that they do not require any intermediary sensory molecules, since these elements themselves behave as sensors transmitting information/action to the gene expression machinery attached to them. Riboswitches are specific noncoding parts of mRNAs that contain ligand-binding moieties, and have the ability to function as genetic switches, inducing a conformational change in the expression platform allowing the modulation of downstream expression events [17]. Currently, more than 40 distinct classes are described [18]. Riboswitches may be formed by in tandem structures, and, in bacteria, are *cis-*regulation elements located in the 5’ untranslated region of mRNAs (5’UTR), where they recognize small molecules assigned as ligands [19,20]. They are basically constituted by two functional domains: the aptamer domain, which function as the receptor for small molecules and, the expression platform, which contains a secondary structural switch that drives the gene expression machinery. The riboswitch used in the present study was the chimera theo/*metE* described by Ceres et al. [19], which is able to control gene expression at the transcriptional level (Figure 1). The aptamer “theo” recognizes the small metabolite theophylline, which is structurally related to caffeine, and used as a bronchodilator [21]. The expression platform *metE*, derived from *Bacillus subtilis* (*B. subtilis*), is an OFF switch that terminates transcription upon binding of the effector ligand.

Here we propose the functional application of the riboswitch theo/*metE* as a novel genetic tool for gene expression modulation in *X. citri*. To evaluate its effectiveness, the riboswitch theo/*metE* was inserted at the 5’-end of the *parB* gene of *X. citri* with the help of the suicide plasmid pNPTS138, which has been extensively used for gene deletion in many Gram-negative bacteria [6,22,23,24,25,26,27]. The expression of *parB* was monitored using qRT-PCR, which showed that the riboswitch theo/*metE* was able to repress 50% of *parB* transcripts. Finally, we followed the ParB-GFP dynamics in *X. citri* cells using fluorescence microscopy, and found that this protein is stable, with a lifetime that certainly spans bacterial generations.

## 2. Material and Methods

### 2.1. Bacterial Strains and Growth Conditions

The bacterial strains and plasmids used in the present work are listed in Table 1. The *E. coli* strain DH10B used for cloning was cultivated in LB/LB-agar (Luria Bertani [28]) at 37 °C. *Xanthomonas citri* subsp. *citri* (*X. citri*) strain 306 was cultivated at 30 °C in NYG rich medium (Nitrogen/Yeast/Glycerol: 5 g/L peptone, 3 g/L yeast extract, 20 g/L glycerol, pH 7.0) or in NYG-agar plates (NYG medium containing agar 15 g/L). The antibiotics ampicillin and kanamycin were used when required at the concentration of 20 μg/mL.

### 2.2. Theophylline Stock Solution

Theophylline (1,3-Dimethylxanthine, 2,6-Dihydroxy-1,3-dimethylpurine, 3,7-Dihydro-1,3-dimethyl-1*H*-purine-2,6-dione) was obtained from Sigma-Aldrich, St. Louis, USA (T1633). A 46 mM stock solution was prepared by dissolving theophylline in nearly-boiling deionized water. Solution was sterilized by filtration through a 33 mm syringe filter with a 0.22 µm pore size hydrophilic polyethersulfone (PES) membrane.

### 2.3. Molecular Biology Procedures

**Figure 2:**
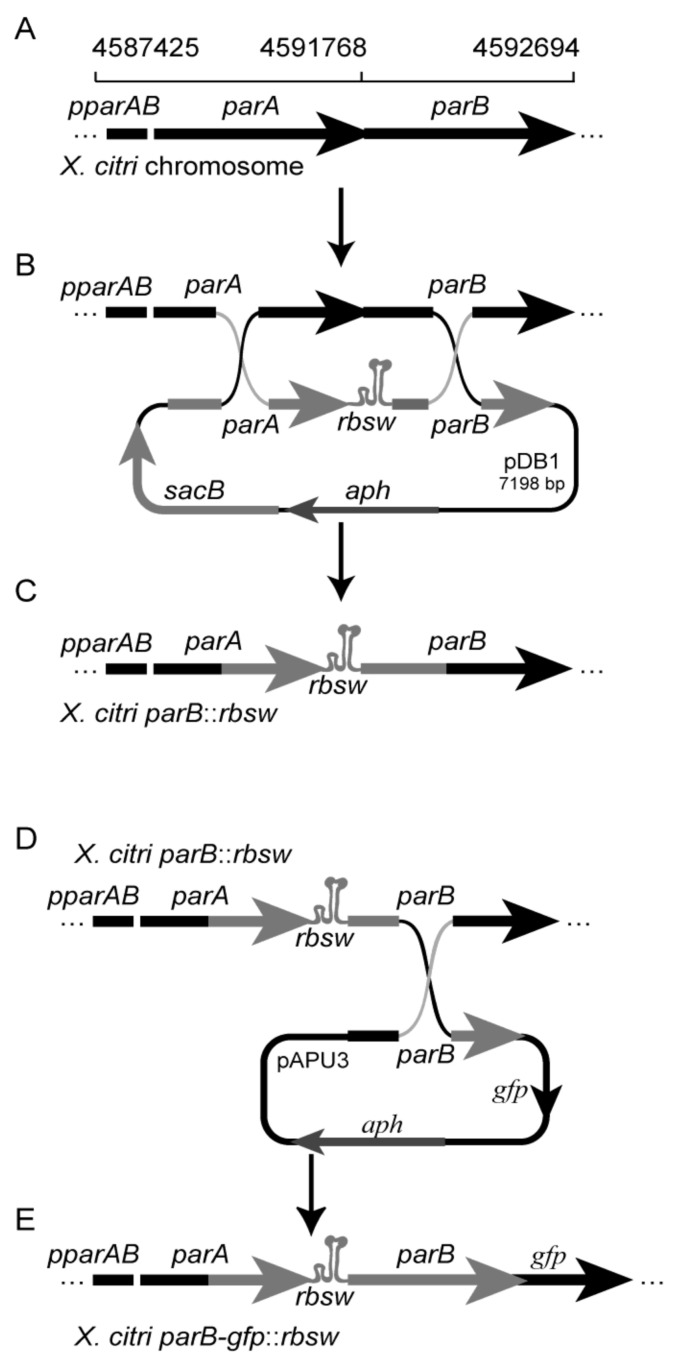
Schematic representation of the genomic region of *X. citri* carrying the riboswitch theo*/metE* and the mutant *X. citri parB-gfp*::*rbsw*. (**A**) Structure and genomic coordinates of the *parA*B operon of *X. citri* strain 306. (**B**) The riboswitch theo*/metE* was inserted between the *parA* and *parB* genes by a double crossover between plasmid pDB1 and the bacterial chromosome, giving rise to the genomic structure shown in (**C**) for the strain *X. citri parB*::*rbsw*. (**D**) The double crossover between plasmid pAPU3 and the chromosome of *X. citri parB*::*rbsw*, giving rise to the genomic structure shown in (**E**) for the strain *X. citri parB-gfp*::*rbsw*, in which the expression of ParB-GFP can be modulated by the riboswitch theo*/metE*.

General molecular biology procedures followed [28]. Polymerases, restriction, and modification enzymes were purchased from Thermo Fisher Scientific, Waltham, USA. The oligonucleotides sequences are shown in the (Supplementary Material Table S1). Riboswitch theo/*metE*: the dsDNA fragment coding for the riboswitch theo/*metE* was constructed by the hybridization of the oligonucleotides theo/*metE* top and theo/*metE* bottom as follows: oligos were dissolved in TE buffer (10 mM Tris-HCl, and 1 mM EDTA pH 8.0) to 100 mM, and mixed in equimolar amounts in a total volume of 10 μL. The mixed oligonucleotides were heated to 90 °C for 15 min using a dry thermal blot and subsequently let to cool gently until reaching room temperature. The product of the hybridization reaction was digested using the restriction enzymes *Xba*I/*Nde*I for further ligation. Sequencing of pNPTS138: the integrative plasmid pNPTS138 (a gift from Professor Lucy Shapiro, Department of Developmental Biology, Stanford University, USA) was sequenced using Sanger Technology (GenBank Accession number: MK533795) (Figure S1). Construction of pDB1: the DNA fragments corresponding to *parA* gene (*X. citri* genomic coordinates 4590983.4591768) and *parB* gene (4591768.4592311) [29] were amplified by PCR using Pfu DNA polymerase and the oligonucleotides 201409 *parA*F/201409 *parA*R and 201409 *parB*F/20140220 *parB*R, respectively. The *parA* and *parB* PCR products were subsequently digested with the enzymes *Bam*HI/*Xba*I and *Nde*I/*Hind*III, respectively, and purified from the agarose gel. The fragments *parA* and *parB* along with the dsDNA riboswitch theo/*metE* were ligated into pNPTS138 linearized with the enzymes *Bam*HI/*Hind*III, giving rise to pDB1. All the amplification products were checked by DNA sequencing at Macrogen (Korea). Construction of the *X. citri* mutants: *X. citri* was transformed by electroporation [31] using the suicide plasmid pDB1 in order to integrate the riboswitch theo/*metE* between the *parA*/ *parB* genes, which gave rise to *X. citri parB::rbsw* (Figure 2A–C). A detailed protocol for the allele exchange method used can be found in [6]. The mutant *X. citri parB-gfp::rbsw* was also generated by a double crossover event by the transformation of *X. citri parB::rbsw* with the suicide plasmid pAUP3 [30] (Figure 2D,E).

### 2.4. Compound Susceptibility and Cell Viability Analyses

The ability of theophylline to inhibit *X. citri* growth was monitored by the Resazurin Microtiter Assay (REMA) essentially as described by [32]. The concentration range of theophylline tested was 0.25 to 32 mM. Development of the test was carried out using the plate reader Synergy H1N1 (BioTek). To define the minimal bactericidal concentration (MBC) of theophylline able to kill *X. citri*, samples from the REMA assay were collected using a 96-wells plate replicator (Sigma-Aldrich, St. Louis, MO, USA), previously to the addition of resazurin (Sigma-Aldrich, St. Louis, MO, USA), and inoculated on NYG-agar plates. Plates were incubated at 30 °C for up to 72 h to allow bacterial growth after theophylline exposure. For the in vitro growth curves, *X. citri* and *X. citri parB::rbsw* were cultivated in NYB medium for 14 h at 30 °C and 200 rpm. Cultures were diluted in fresh NYB medium to an OD600nm of 0.1 in a final volume of 1.5 mL (OD600nm of 0.3 corresponds to 10^8^ CFU/mL). Cell cultures were distributed in the wells of a 24-wells microtiter plate, theophylline was added as needed to a final concentration of 2 mM and the plates were incubated in the plate reader Synergy H1N1 (BioTek) at 30 °C with constant agitation (200 rpm) and OD600nm were measured every 30 min for 20 h [6]. Three independent experiments of REMA, MBC, and growth curves were performed in triplicates each. GraphPad Prism version 6 was used for the statistical analyses.

### 2.5. Fluorescence Microscopy

The mutant *X. citri parB-gfp::rbsw* was cultivated in 5.0 mL of NYG medium for approximately 16 h at 30 °C and 200 rpm. The cultures were adjusted to the OD600nm of 0.1 with fresh NYG medium for a final volume of 5.0 mL and subsequently cultivated in the same conditions for approximately four hours. At this point, theophylline was added to the medium to a final concentration of 2 mM and the culture was kept at 30 °C and 200 rpm to monitor the ParB-GFP dynamics within the cells. Negative controls without the addition of theophylline were also prepared in the same growth conditions as described above. At the points of 4 h (T0 of theophylline exposure), 6 h (2 h of theophylline exposure), 8 h (4 h of theophylline exposure), and 16 h (12 h of theophylline exposure) drops of 5 µL of cell culture were placed on microscope slides prepared for microscope imaging as described by [33]. Bacteria were visualized using an Olympus BX61 microscope equipped with a monochromatic camera OrcaFlash2.8 (Hamamatsu, Japan) and GFP filter. Data collection and analysis were performed with the software CellSens Version 11 (Olympus, Hamburg, Germany).

### 2.6. Real-Time Reverse Transcription PCR (qRT-PCR)

The expression of *parB* was evaluated by qRT-PCR analysis using *X. citri* and *X. citri parB::rbsw*. Bacterial cells were cultivated in NYG medium supplemented or not with theophylline 2 mM, following the growth condition previously described. To obtain *X. citri parB::rbsw* and *X. citri* 306 total RNA, 1 mL of each bacteria culture (with and without theophylline) was collected at three different time points and pelleted by centrifugation (12,000× *g*) for 10 min using a microcentrifuge. The RNA extraction was done using the RNA Plus Mini Kit (QIAGEN, Hilden, Germany) as described by the manufacturer. The first strand of complementary DNA was synthesized from 400 ng of total RNA using the Revert Aid H Minus First Strand cDNA Synthesis Kit (QIAGEN, Hilden, Germany). The qRT-PCR was run on a StepOne Real Time PCR System (Life Technologies, Carlsbad, CA, USA) using the SYBR Green/ROX qPCR Master Mix (2×) (FERMENTAS/Thermo Fisher Scientific, Waltham, MA, USA) and the primers for the gene *parB* (*parB*-RT-F and *parB*-RT-R) and the constitutive gene *rop*B as a control (*rpo*BF and *rop*BR) [34] (Table S1). The fold change of expression of the target genes was obtained using the Applied Biosystems StepOnePlus^TM^ software and calculated using the GraphPad Prism version 6, applying ANOVA parametric test [35,36].

## 3. Results

### 3.1. pNPTS138: A Tool for Allele Exchange in Gram-Negative Bacteria

To facilitate the construction of the integrative plasmid pDB1, we decided to carry out the whole nucleotide sequencing and annotation of the suicide plasmid pNPTS138 (Figure S1). The annotation identified three genes (*traJ*, *aph*, and *sacB*), and a total G + C content of 47.27% in a molecule of 5361 bp. The multiple cloning site (polylinker) available in pNPTS138 was extracted from the commercial vector pLITMUS38i (New England Biolabs). Therefore, pNPTS138 also carries the gene fragment *lac*Zα, which enables white-blue screening for the selection of bacterial colonies after transformation into the cloning strain. It harbors the transcriptional regulator gene *tra*J, which is involved with the conjugal transfer of DNA between bacteria. As a primary selective marker, it carries the aminoglycoside 3′-phosphotransferase (*aph*) gene 99.3% identical to the *E. coli aphA1* gene (UniProt: P00551), which confers resistance to the antibiotic Kanamycin and structurally related aminoglycosides, including amikacin. Finally, pNPTS138 has the *sac*B gene from *Bacillus subtilis*, which encodes for levansucrase, an enzyme that metabolizes sucrose to levans that are toxic to Gram-negative bacteria.

### 3.2. Effect of Theophylline Concentration on X. citri Growth

**Figure 3:**
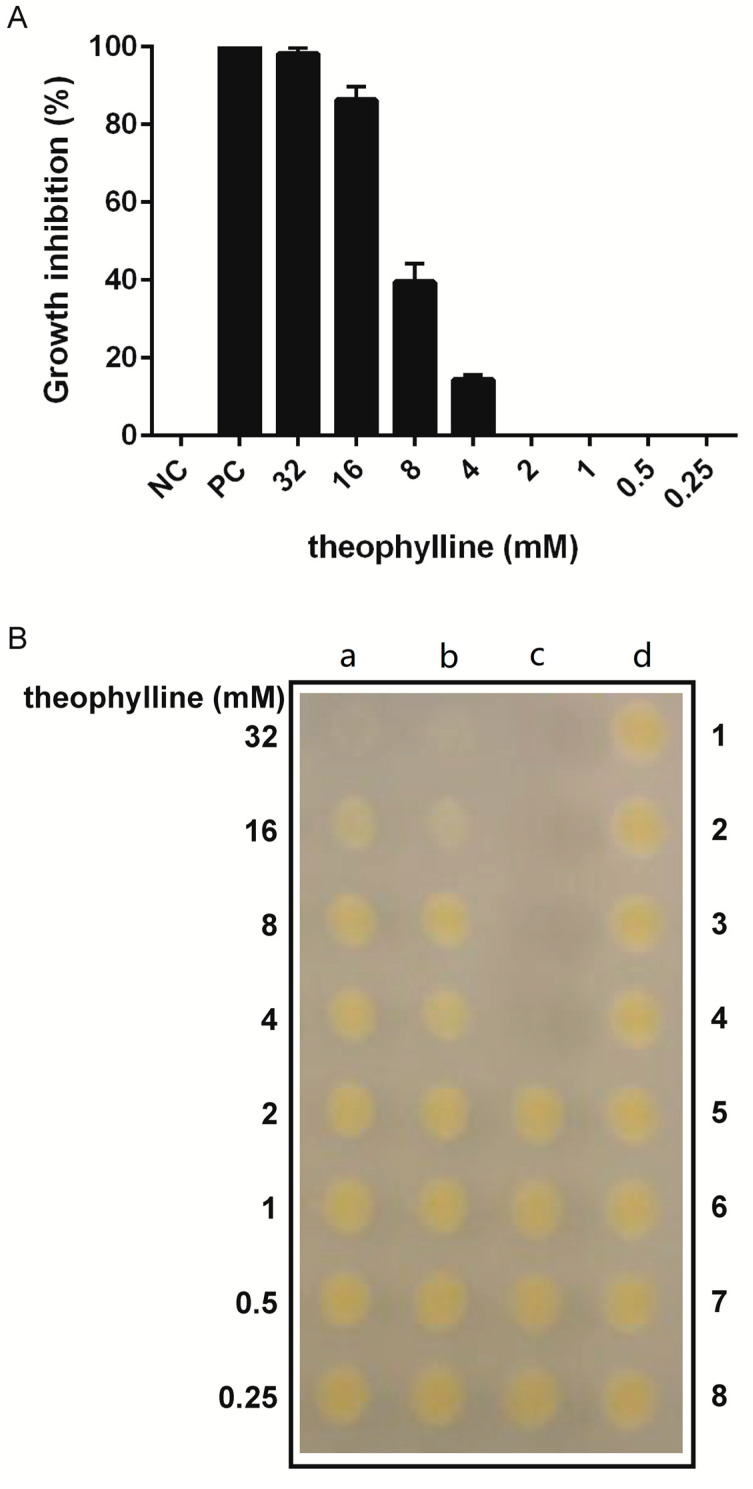
*Xanthomonas citri* growth inhibition induced by theophylline. Bacteria were exposed to theophylline at the concentration range varying from 0.25 to 32 mM using a Resazurin Microtiter Assay (REMA) setting. Growth inhibition was determined by monitoring the cell respiratory activity using resazurin (**A**), and the ability to resume growth after treatment (bacteriostatic/bactericidal effect) was investigated by cell plating (**B**). (**A**) Dose-response data derived from the growth inhibition experiment. Bars indicate the average percentage of growth inhibition, while lines above the bars represent the standard deviation of the means. NC: untreated cells; PC: positive control kanamycin at 20 mg/mL. (**B**) Samples were collected from REMA and inoculated on NYG-agar plates, without theophylline, to evaluate the ability of the cells to resume growth. (a) 1–8 and (b) 1–8 illustrate a replica experiment in which cells were exposed to theophylline at the concentrations indicated; (c) 1–4, positive control treated with kanamycin at 20 mg/mL; (c) 5–8, vehicle control, water used to dissolve theophylline, and (d) 1–8, negative control, untreated cells. All data were derived from three independent experiments.

The wild type *X. citri* (strain 306) was cultivated in the presence of various concentrations of theophylline and its toxicity was monitored using REMA, which measures bacterial respiration (Figure 3A). We observed a clear dose-dependent effect related to theophylline for concentrations above 4 mM, which had an influence on bacterial growth. Eight millimolar theophylline was enough to inhibit practically 40% of the bacterial cells in the culture. At the maximum concentration used of 32 mM, *X. citri* growth was almost completely inhibited (nearly 100%), reaching inhibition levels comparable to our positive control, the antibiotic kanamycin at 20 g/mL. In our analyses, 2 mM theophylline was unable to inhibit *X. citri* growth.

Together with REMA, we also assessed the ability of *X. citri* to resume growth after exposure to theophylline (Figure 3B). According to the results, theophylline at the concentration of 32 mM exhibited a bactericidal effect, where cells were unable to resume growth upon plating them on non-selective NYG-medium following treatment with the compound. Although the exposure to theophylline at the concentrations of 16 mM, 8 mM, and 4 mM led to the inhibition of bacterial respiration, the effect was only bacteriostatic, and cells could grow normally on plate after treatment.

**Figure 4:**
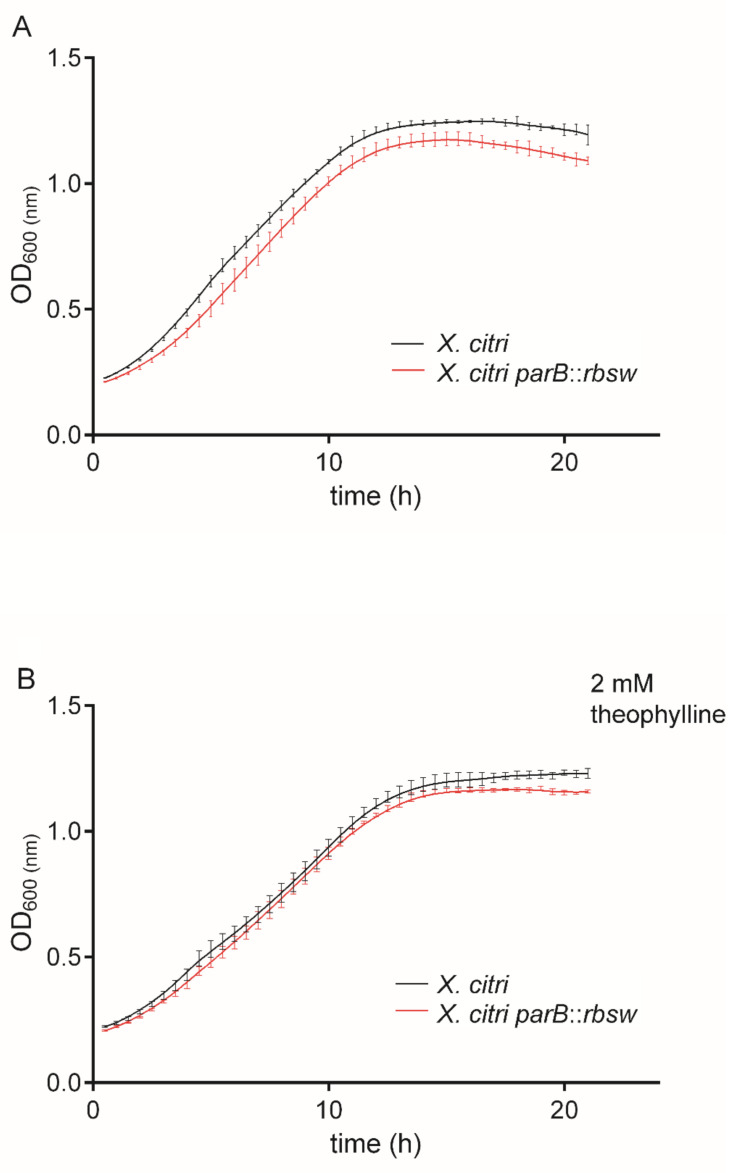
*Xanthomonas citri* mutant carrying the riboswitch theo*/metE* showed a normal growth pattern. Wild type *X. citri* and *X. citri parB*::*rbsw* were cultivated in NYG medium from the OD600nm of ~0.1 in the absence (**A**) and presence (**B**) of 2 mM theophylline. Growth was monitored by OD600nm measurements taken every 30 min for a total period of 20 h. Here we show a representative experiment in which the points in the curves are the averages of triplicate cultures, and the vertical lines correspond to the standard deviation values of the means. Three independent experiments were performed.

Once settled that 2 mM theophylline was the concentration that did not affect *X. citri* growth, we further investigated theophylline toxicity by long term exposure of the wild type and mutant *X. citri parB::rbsw* (carrying the riboswitch theo/*metE* integrated between *parA* and *parB*) to 2 mM theophylline in growth curves (Figure 4). Both in vitro tests (in the presence and absence of theophylline) showed that the strains grew equally well for the time-course monitored with no detectable differences between wild type *X. citri* and mutant *X. citri parB::rbsw.* Both strains followed practically the same growth pattern in the lag and log-phases. Therefore, data confirmed that the genetic alteration induced by the integration of the riboswitch into *X. citri* chromosome did not affect bacterial fitness that could interfere on subsequent evaluations of the riboswitch function.

### 3.3. Theophylline Influences the Expression of parB

**Figure 5:**
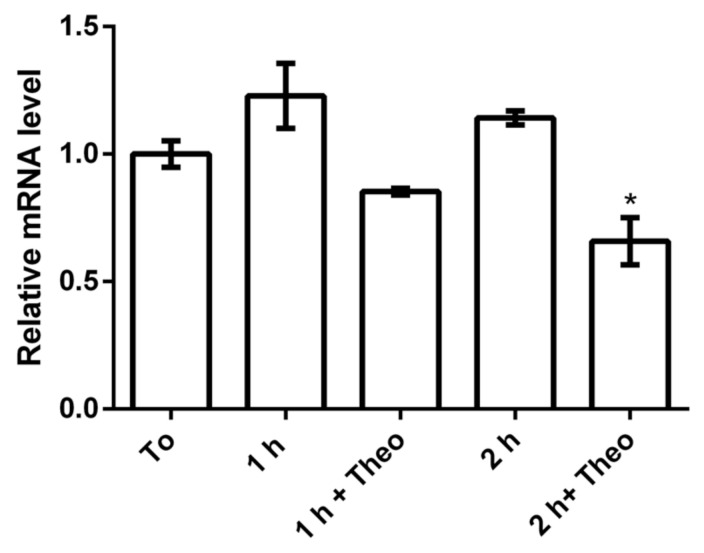
Effect of theophylline on the expression of *parB*. The mutant strain *X. citri parB*::*rbsw* was cultivated from the starting OD600nm of ~0.1 for a total period of 4 h (T0). Theophylline was added to a final concentration of 2 mM, and the *parB* mRNA was monitored by qRT-PCR. T0: *parB* mRNA expression level after 4 h of bacterial growth; 1 h: untreated after 5 h of growth; 1 h + theo: 1 h of growth under exposure to 2 mM theophylline, total growth of 5 h; 2 h: untreated after 6 h of growth, and 2 h + theo: 2 h of growth under exposure to 2 mM theophylline, total growth of 6 h. * indicates significant difference according to ANOVA test.

The functionality of the riboswitch theo/*metE* was first investigated by monitoring the expression of *parB* in the mutant *X. citri parB::rbsw* exposed or not to theophylline. The expression of *parB* was checked by assessing the *parB* mRNA levels using qRT-PCR (Figure 5). The qRT-PCR assay showed that the *parB* mRNA level decreased as the time of exposure to theophylline increased. The level of the *parB* transcript was suppressed by theophylline with a 2-fold change by the time of 2 h of exposure as compared with the same 2 h in the absence of the compound. These results confirm that there is a discrete influence of theophylline in the expression of *parB* mRNA probably influenced by the concentration of mRNA and the exposure timeframe to theophylline.

### 3.4. Chromosome Segregation Is Not Affected by the Downregulation of parB Expression

**Figure 6:**
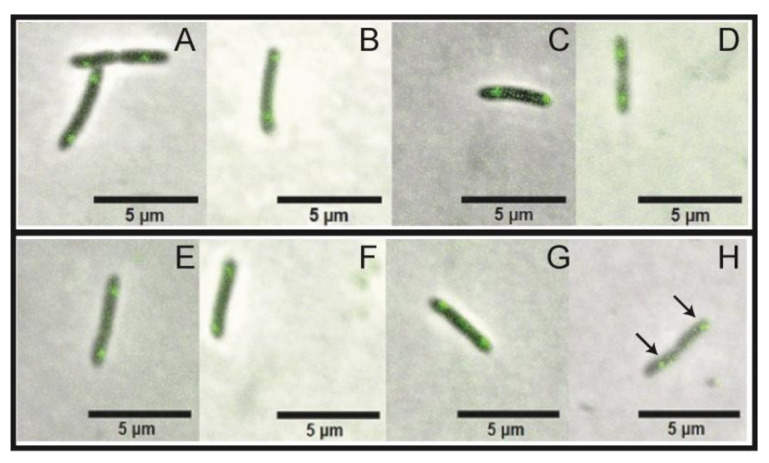
Time-course of *X. citri parB-gfp::rbsw* showing the localization of ParB-GFP. Absence of theophylline photographed at 4 h, 6 h, 8 h, and 16 h, (**A**–**D**), respectively. Panels (**E**–**H**) theophylline was added to the cultures after 4 h of growth, and cells were imaged at the time points of 4 h (T0 of theophylline exposure (**E**)), 6 h (2 h of theophylline exposure (**F**)), 8 h (4 h of theophylline exposure (**G**)) and 16 h (12 h of theophylline exposure (**H**)). Scale bar 5 μm. Magnification 100×. Merge of phase contrast and GFP channels. Black arrows indicated the site of ParB-GFP localization.

To evaluate if the *parB* silencing effect induced by the action of the riboswitch theo/*metE* could interfere in chromosome segregation, we used a *X. citri* mutant expressing the segrosome marker ParB-GFP in order to monitor the formation and dynamics of the bacterial centromere. The subcellular localization of ParB-GFP in *X. citri parB-gfp::rbsw* followed the same pattern described by [30]. ParB-GFP was mostly seen as one fluorescent focus per rod, and occupying the region between the center and approximately 2/4 from the old-pole of the rods, which becomes more evident when a division constriction can be seen between cells (Figure 6H, arrows). No difference could be detected in the ParB-GFP fluorescence signal, as well as in the distribution dynamics of ParB-GFP foci during the time-course of exposure to 2 mM theophylline (Figure 6E–H). Here, cells were cultivated from an OD600nm 0f ~0.1 for 4 h (6E), and after exposed to the compound for 2 h (6F), 4 h (6G), and 12 h (6H). Note that even after a long exposure to 2 mM theophylline (12 h; Figure 6H) the presence of theophylline did not affect the bacterial chromosome segregation process.

## 4. Discussion

The nucleotide sequence of the plasmid pNPTS138 was determined, and it was first reported as a public tool in this work. The plasmid developed by the Shapiro’s group has key characteristics that enable easy gene cloning, as well as the integration or deletion of genetic markers into bacterial chromosomes by using the allele exchange technique [6,22,23,25]. Among these features, pNPTS138 is replicative in *E. coli* and a suicide vector in many other Gram-negative bacteria, including *X. citri* [24,26,27,37]. In addition to its antibiotic resistance marker, pNPTS138 harbors the *sacB* gene from *B. subtilis*, which offers an easy and effective counterselection method during the two-step allele exchange protocol. Briefly, cells are transformed with pNPTS138-based constructs, and those in which the plasmid became integrated into the bacterial chromosome can be selected on media supplemented with kanamycin (see our illustration in Figure 2B). The first crossover event may happen between *parA* or *parB* in the plasmid and the respective homologous region in the chromosome. Next, upon the occurrence of the second recombination event between the other plasmid region not involved in the first event and its homologue in the chromosome, the plasmid will be released with the conclusion of the allele exchange process. Cells in which the double crossover events took place can be identified by plating on media supplemented with sucrose. The enzyme levansucrase, encoded by *sacB*, is inducible in the presence of sucrose, which leads to the synthesis of lethal amounts of levans and their accumulation in the Gram-negative bacterial periplasm leading to cell lysis [38]. A detailed description of the use of pNPTS138 for gene deletion in *X. citri* was described in [6].

In the present work, pNPTS138 was used to deliver the riboswitch theo/*metE* into *X. citri* chromosome (an integration and not a deletion event) in order to evaluate its effectiveness for gene transcription control in this plant pathogen. The riboswitch theo/*metE* is a chimeric tool developed by the fusion of the theophylline aptamer domain with the *metE* expression platform, which is intended to control gene expression at the transcriptional level [19]. When placed right upstream of a gene, the riboswitch is transcribed with it, becomes part of its mRNA, and has the potential to control its transcription/translation (Figure 1). In the particular case of the riboswitch theo/*metE*, theophylline binding to the aptamer moiety right after the transcription of the riboswitch induces the formation of a structure that aborts the progress of transcription to the 3′-end of it (OFF mode). To validate its function, the riboswitch theo/*metE* was integrated just upstream of the *X. citri parB* gene. The protein ParB is responsible for the assembly of the bacterial centromere that coordinates chromosome segregation in many bacteria [7].

We showed that in the presence of 2 mM theophylline, *parB* mRNA was repressed in a two-fold ratio (nearly 50% of transcriptional repression). For *B. subtilis* a β-galactosidase repression of 8-fold was obtained when 6 mM theophylline was used [39]. Even though the theo/*metE* chimera showed a >90% repression at higher theophylline concentrations [19], we decided to perform our assays with *X. citri* at 2 mM theophylline. The reason for this is that in our dose-response analyses, 2 mM theophylline was defined as a safe dose that did not interfere with *X. citri* growth. Low theophylline concentrations surely decreased the efficiency of the riboswitch theo/*metE* in gene repression, but it also prevented interferences in bacterial fitness. Indeed, we showed here that long-term exposure to the ligand had no apparent effect on fitness, where mutant and wild type *X. citri* strains had similar performances over the course of 20 h of exposure to 2 mM theophylline (see Figure 4). There are other factors that could interfere with the function of the riboswitch theo/*metE* and that may explain the efficiency of 50% transcriptional repression we achieved in this work. As an example, it was shown that the regulation of gene expression by the theophylline-inducible riboswitch is influenced by the concentration of mRNA [40]. In such case, the target gene to be controlled might respond differently to the riboswitch depending on its transcriptional levels. Further comparative analyses are needed to clarify this behavior.

The sub-cellular localization of ParB-GFP was subsequently used to investigate the downstream effects of *parB* gene transcription silencing in *X. citri*. The ParB-GFP fusion protein has a clear detectable distribution pattern within cells under the microscope, and disruption and/or deletion of ParB generates *X. citri* cells with increased size, which may show interference with nucleoid organization [6,30]. Although we could not detect any difference in the ParB-GFP dynamics displayed by *X. citri parB-gfp::rbsw* when exposed or not to theophylline, there is no available information on the period that the ParB protein stays functional within *X. citri*. According to previous results from [6], ParB seems stable for many bacterial generations. This observation corroborates early studies of the ParB dynamics in *B. subtilis*, in which clearly ParB-GFP foci split during chromosome replication and segregation, and could well end up in the daughter cells lately upon cell division [41]. This idea is supported by the fact that several monomers of ParB are present within the cells to organize the centromere when they bind and polymerize around the replication origin of the bacterial chromosome [42]. Accordingly, [8], analyzing more than a thousand cells of *Vibrio cholerae,* showed that, around the time of division, the ParB polar signal in a cell compartment duplicates, and one of them segregates across the cell towards the opposite pole. When Vibrio cholerae divides, each of the poles/ParB-focus will be inherited by a distinct daughter cell. Based on that, ParB seems indeed a stable protein that is transferred from parental to daughter cells, which makes it difficult to be depleted, and perhaps to have cells void of them. In addition, our system showed a 50% efficiency as it is, therefore, if a small amount of protein is still being produced, they will reinforce the presence of ParB-GFP in our analyses. We followed the ParB-GFP signal for at least six bacterial generations, and according to our results, the presence of ParB was stable.

Even though we have achieved just a modest repression of *parB*, our experiments showed the responsiveness of the system in our bacterial model, and confirmed its functionality in *X. citri*. Further studies involving nucleotide sequence optimization and/or combination of ligand binding moieties with different expression platforms intended to improve the structure of the riboswitch could lead to higher efficiencies in transcription control, such as reported by [19,43]. The optimization of the riboswitch response has been used for *B. subtilis metE*, *yitJ* and *lysC* expression platforms throughout the introduction of a set of mutation on the 5’-side of the riboswitch [19], a strategy that could also be applied for future studies exploring the use of synthetic riboswitches in *Xanthomonas*.

The strategy reported here to control gene expression in *X. citri* is new. The integration of the riboswitch into *X. citri* chromosome may have changed the genetic structure of the region around the site of integration, but it did not affect bacterial fitness nor generated detectable phenotypic changes in the absence of theophylline (OFF mode). This is remarkable and differs enormously from the standard genetic methods in which DNA deletion or gene disruption are used to study gene function. Simple deletion and disruption of *parB* had detectable phenotypes in *X. citri* [6,30], and in the past, we had difficulties to delete *parB* from *X. citri*, and that was probably due to the fact that a gene whose product operates in an essential process, such as chromosome segregation, cannot be easily deleted [30]. The integration of a genetic tool that can potentially be used to switch gene expression ON/OFF is an attractive alternative to circumvent such a problem.

## Figures and Tables

**Table 1:** List of strains and plasmids used in this work.

Strains	Characteristics	References
*X. citri* 306	*Xanthomonas citri* subsp. *citri* strain 306 (wild type strain), Ap^R^	IBSBF 1594 [29]
*E. coli* DH10B	Cloning strain	Invitrogen, Thermo Fisher Scientific, Waltham, USA
*X. citri parB::rbsw*	*parA-rbsw-parB*; Ap^R^	This work
*X. citri parB-gfp::rbsw*	*parA-rbsw-parB-gfpmut1*; Ap^R^ and Km^R^	This work
**Plasmids**		
pNPTS138	ColE1-like *ori, sacB, aph*	This work (GenBank MK533795)
pDB1	pNPTS138 carrying the riboswitch theo/*metE* located between *parA* (4590983.4591768) * and *parB* (4591768.4592311) * = *parA-rbsw-parB*	This work
pAPU3	*xylR, pxyl, parB-gfpmut1, bla, aph*	[30]

Ap^R^ and Km^R^, ampicillin and kanamycin resistance, respectively; *bla* and *aph*, genes for beta-lactamase (Ap^R^) and aminoglycoside phosphotransferase (Km^R^), respectively. * genomic coordinates according to [29].

## Data Availability

The data presented in this study are available in the Supplementary Material.

## Supplementary Materials

The following are available online at https://www.mdpi.com/2076-2607/9/2/329/s1.
