# Supplementary material for: Riboswitch theo/metE as a Transcription Regulation Tool for Xanthomonas citri subsp. citri
Source: Microorganisms. 2021 Feb 6;9(2):329. doi: 10.3390/microorganisms9020329 (PMC7914508; doi:10.3390/microorganisms9020329)
Supplement: Supplementary file 1 [file microorganisms-09-00329-s001.pdf]

Supplementary material:

Riboswitch theo/*metE* as a transcription regulation tool for *Xanthomonas citri* subsp. *citri*

Danilo Bueno, Danielle B. Pedrolli, Paula M. M. Martins, Daniela A. Bocchini, Karen C. M. Moraes, Agda P. Facincani, Jesus A. Ferro, Alessandro M. Varani, Michelle M. Pena, Henrique Ferreira

Table S1: Oligonucleotides designed for the construction of the riboswitch and PCR amplification

Name	Sequence
**theo/*met*E top**	5'TCTAGACAAAAAATTAATAACATTCTCTCTTAATACCAGCTTCGAAAGAAGC CCTTGGCAGTGAGAGAGGCAGTGTTTTACGTAGAAAAGCCTCTTTCTCTCATGG GAAAGAGGCTTTTTGTTGTAGGAGGTTATTCATATG
**theo/*met*E bottom**	5'CATATGAATAACCTCCTACAACAAAAAGCCTCTTTCCCATGAGAGAAAGAGG CTTTTCTACGTAAAACACTGCCTCTCTCACTGCCAAGGGCTTCTTTCGAAGCTG GTATTAAGAGAGAATGTTATTAATTTTTTGTCTAGA
**201409 *par*AF**	5' AAGGATCCATCGCCATTGCCAACCAGAAG
**201409 *par*AR**	5' TGTCTAGATTAGACGGTCTCCACGGGCC
**201409 *par*BF**	5' TTCATATGAACAAGCCGATCCCCGCAAAG
**20140220 *par*BR**	5′ AAAAAGCTTTTAGCTACGCTGCATGCGCAGGC
***par*B-RT-F**	5' GGGCGTGATCCAGCCGATC
***par*B-RT-R**	5' AGCCCAGCCAGCTGCGAAG
***rpo*BF**	5' GGATTCCTATCGCGAATTCCT
***rpo*BR**	5' TGTAGCTGGAAATCGGGAACA

The sites for the restriction enzymes used are underlined.

Figure S1. Map of the cloning vector pNPTS138 (Genbank MK533795)

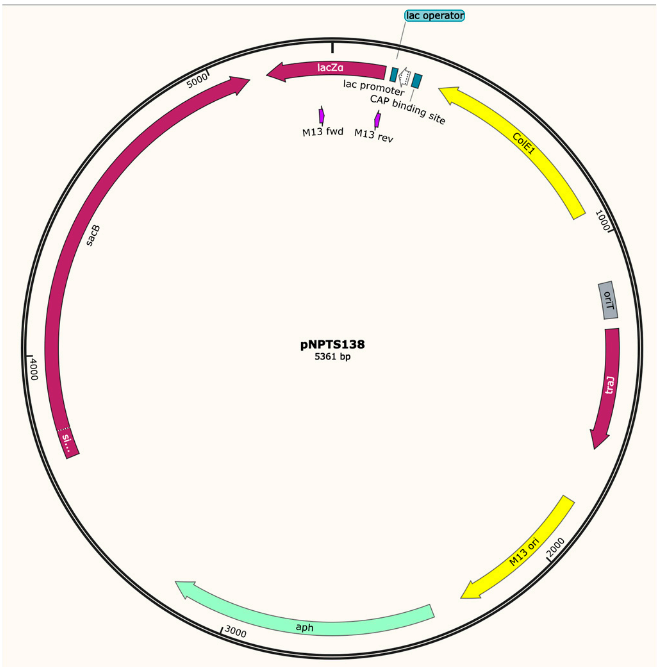


```
LOCUS       MK533795                5361 bp    DNA     circular SYN 17-JUL-2019
DEFINITION  Cloning vector pNPTS138 plasmid pNPTS138, complete sequence.
ACCESSION   MK533795
VERSION     MK533795.1
KEYWORDS    .
SOURCE      Cloning vector pNPTS138
  ORGANISM  Cloning vector pNPTS138
            other sequences; artificial sequences; vectors.
REFERENCE   1  (bases 1 to 5361)
  AUTHORS   Ferro,J.A., Facincani,A.P., Tezza,R.D., Varani,A.M. and Ferreira,H.
  TITLE     The nucleotide sequence of the plasmid pNPTS138
  JOURNAL   Unpublished
REFERENCE   2  (bases 1 to 5361)
  AUTHORS   Ferro,J.A., Facincani,A.P., Tezza,R.D., Varani,A.M. and Ferreira,H.
  TITLE     Direct Submission
  JOURNAL   Submitted (18-FEB-2019) Departamento de Tecnologia, Univ. Estadual
            Paulista 'Julio de Mesquita Filho' - UNESP-Jaboticabal, Via de
            Acesso Prof.Paulo Donato Castellane s/n, Jaboticabal, SP 14884-900,
            Brazil
COMMENT     The plasmid was a gift from Prof. Lucy Shapiro, Department of
            Developmental Biology, Stanford University School of Medicine
            Beckman Center 279 Campus Drive, B300 Stanford, CA 94305.

            ##Assembly-Data-START##
            Sequencing Technology :: Sanger dideoxy sequencing
            ##Assembly-Data-END##
FEATURES             Location/Qualifiers
     source          1..5361
                     /organism="Cloning vector pNPTS138"
                     /mol_type="other DNA"
                     /db_xref="taxon:2592094"
                     /plasmid="pNPTS138"
     gene            1282..1653
                     /gene="traJ"
     CDS             1282..1653
                     /gene="traJ"
                     /note="Protein TraJ"
                     /codon_start=1
                     /transl_table=11
                     /product="TraJ"
                     /protein_id="QDK64793.1"
                     /translation="MADETKPTRKGSPPIKVYCLPDERRAIEEKAAAAGMSLSAYLLA
                     VGQGYKITGVVDYEHVRELARINGDLGRLGGLLKLWLTDDPRTARFGDATILALLAKI
                     EEKQDELGKVMMGVVRPRAEP"
```

```
     gene            2371..3186
                     /gene="neo"
     CDS             2371..3186
                     /gene="neo"
                     /EC_number="2.7.1.95"
                     /note="Aminoglycoside 3'-phosphotransferase"
                     /codon_start=1
                     /transl_table=11
                     /product="Neo"
                     /protein_id="QDK64794.1"
                     /translation="MSHIQRETSCSRPRLNSNMDADLYGYKWARDNVGQSGATIYRLY
                     GKPDAPELFLKHGKGSVANDVTDEMVRLNWLTEFMPLPTIKHFIRTPDDAWLLTTAIP
                     GKTAFQVLEEYPDSGENIVDALAVFLRRLHSIPVCNCPFNSDRVFRLAQAQSRMNNGL
                     VDASDFDDERNGWPVEQVWKEMHKFLPFSPDSVVTHGDFSLDNLIFDEGKLIGCIDVG
                     RVGIADRYQDLAILWNCLGEFSPSLQKRLFQKYGIDNPDMNKLQFHLMLDEFF"
     gene            3686..5107
                     /gene="sacB"
     CDS             3686..5107
                     /gene="sacB"
                     /EC_number="2.4.1.10"
                     /note="Levansucrase"
                     /codon_start=1
                     /transl_table=11
                     /product="SacB"
                     /protein_id="QDK64795.1"
                     /translation="MNIKKFAKQATVLTFTTALLAGGATQAFAKETNQKPYKETYGIS
                     HITRHDMLQIPEQQKNEKYQVPEFDSSTIKNISSAKGLDVWDSWPLQNADGTVANYHG
                     YHIVFALAGDPKNADDTSIYMFYQKVGETSIDSWKNAGRVFKDSDKFDANDSILKDQT
                     QEWSGSATFTSDGKIRLFYTDFSGKHYGKQTLTTAQVNVSASDSSLNINGVEDYKSIF
                     DGDGKTYQNVQQFIDEGNYSSGDNHTLRDPHYVEDKGHKYLVFEANTGTEDGYQGEES
                     LFNKAYYGKSTSFFRQESQKLLQSDKKRTAELANGALGMIELNDDYTLKKVMKPLIAS
                     NTVTDEIERANVFKMNGKWYLFTDSRGSKMTIDGITSNDIYMLGYVSNSLTGPYKPLN
                     KTGLVLKMDLDPNDVTFTYSHFAVPQAKGNNVVITSYMTNRGFYADKQSTFAPSFLLN
                     IKGKKTSVVKDSILEQGQLTVNK"
ORIGIN
        1 tagagggtcg acgcatgcct gtacatccgg agacgcgtca cggccgaagc tagcgaattc
       61 gtggatccag atatcctgca gagaagcttg gcgccagccg gcttcaattg cacgggcccc
      121 actagtgagt cgtattacgt agcttggcgt aatcatggtc atagctgttt cctgtgtgaa
      181 attgttatcc gctcacaatt ccacacaaca tacgagccgg aagcataaag tgtaaagcct
      241 ggggtgccta atgagtgagc taactcacat tacatgtgag caaaaggcca gcaaaaggcc
      301 aggaaccgta aaaaggccgc gttgctggcg tttttccata ggctccgccc ccctgacgag
      361 catcacaaaa atcgacgctc aagtcagagg tggcgaaacc cgacaggact ataaagatac
      421 caggcgtttc cccctggaag ctccctcgtg cgctctcctg ttccgaccct gccgcttac
      481 cggatacctg tccgcctttc tcccttcggg aagcgtggcg ctttctcata gctcacgctg
      541 taggtatctc agttcggtgt aggtcgttcg ctccaagctg ggctgtgtgc acgaaccccc
      601 cgttcagccc gaccgctgcg ccttatccgg taactatcgt cttgagtcca acccggtaag
      661 acacgactta tcgccactgg cagcagccac tggtaacagg attagcagag cgaggtatgt
      721 aggcggtgct acagagttct tgaagtggtg gcctaactac ggctacacta gaagaacagt
      781 atttggtatc tgcgctctgc tgaagccagt taccttcgga aaaagagttg gtagctcttg
      841 atccggcaaa caaaccaccg ctggtagcgg tggttttttt gtttgcaagc agcagattac
      901 gcgcagaaaa aaaggatctc aagaagatcc tttgatcttt tctacggggt ctgacgctca
      961 gtggaacgaa aactcacgtt aagggatttt ggtcatggac ggatcttttc cgctgcataa
     1021 ccctgcttcg gggtcattat agcgattttt tcggtatatc catccttttt cgcacgatat
     1081 acaggatttt gccaaagggt tcgtgtagac tttccttggt gtatccaacg gcgtcagccg
     1141 ggcaggatag gtgaagtagg cccacccgcg agcgggtgtt ccttcttcac tgtcccttat
     1201 tcgcacctgg cggtgctcaa cgggaatcct gctctgcgag gctggccggc taccgccggc
     1261 gtaacagatg agggcaagcg gatggctgat gaaaccaagc caaccaggaa gggcagccca
     1321 cctatcaagg tgtactgcct tccagacgaa cgaagagcga ttgaggaaaa ggcggcggcg
     1381 gccggcatga gcctgtcggc ctacctgctg gccgtcggcc agggctacaa aatcacgggc
     1441 gtcgtggact atgagcacgt ccgcgagctg gcccgcatca atggcgacct gggccgcctg
     1501 ggcggcctgc tgaaactctg gctcaccgac gacccgcgca cggcgcggtt cggtgatgcc
     1561 acgatcctcg ccctgctggc gaagatcgaa gagaagcagg acgagcttgg caaggtcatg
     1621 atgggcgtgg tccgcccgag ggcagagcca tgactttttt agccgctaaa acggccgggg
     1681 ggtgcgcgtg attgccaagc acgtccccat gcgctccatc aagaagagcg acttcgcgga
     1741 gctggtgaag tacatcaccg acgagcaagg caagaccgat cccccatgag attatcaaaa
     1801 aggatcttca cctagatcct tttacgcgcc ctgtagcggc gcattaagcg cggcgggtgt
     1861 ggtggttacg cgcagcgtga ccgctacact tgccagcgcc ctagcgcccg ctcctttcgc
     1921 tttcttccct tcctttctcg ccacgttcgc tttccccgtc aagctctaaa tcgggggctc
     1981 cctttagggt tccgatttag tgctttacgg cacctcgacc ccaaaaaact tgatttgggt
     2041 gatggttcac gtagtgggcc atcgccctga tagacggttt ttcgcccttt gacgttggag
     2101 tccacgttct ttaatagtgg actcttgttc caaactggaa caacactcaa ccctatctcg
     2161 ggctattctt ttgatttata agggattttg ccgatttcgg cctattggtt aaaaaatgag
     2221 ctgatttaac aaaaatttaa cgcgaatttt aacaaaatat taacgtttac aatttctaga
     2281 gcttggccgg gttacattgc acaagataaa aatatatcat catgaacaat aaaactgtct
     2341 gcttacataa acagtaatac aaggggtgtt atgagccata ttcaacggga aacgtcttgc
     2401 tcgaggccgc gattaaattc caacatggat gctgatttat atgggtataa atgggctcgc
     2461 gataatgtcg ggcaatcagg tgcgacaatc tatcgattgt atgggaagcc cgatgcgcca
```

```
2521 gagttgtttc tgaaacatgg caaaggtagc gttgccaatg atgttacaga tgagatggtc
2581 agactaaact ggctgacgga atttatgcct cttccgacca tcaagcattt tatccgtact
2641 cctgatgatg catggttact caccactgcg atccccggta aaacagcatt ccaggtatta
2701 gaagaatatc ctgattcagg tgaaaatatt gttgatgcgc tggcagtgtt cctgcgccgg
2761 ttgcattcga ttcctgtttg taattgtcct tttaacagcg atcgcgtatt tcgtctcgct
2821 caggcgcaat cacgaatgaa taacggtttg gttgatgcga gtgattttga tgacgagcgt
2881 aatggttggc ctgttgaaca agtctggaaa gaaatgcata agtttttgcc attctcaccg
2941 gattcagtcg tcactcatgg tgatttctca cttgataacc ttatttttga cgaggggaaa
3001 ttaataggtt gtattgatgt tggacgagtc ggaatcgcag accgatacca ggatcttgcc
3061 atcctatgga actgcctcgg tgagttttct ccttcattac agaaacggct ttttcaaaaa
3121 tatggtattg ataatcctga tatgaataaa ttgcagtttc atttgatgct cgatgagttt
3181 ttctaatcag aattggttaa ttggttgtaa cactggcaga gcattacgct gacttgacgg
3241 gacggcgccg gccaagctct attatttagt gaaatgagat attatgatat tttctgaatt
3301 gtgattaaaa aggcaacttt atgcccatgc aacagaaact ataaaaaata cagagaatga
3361 aaagaaacag atagattttt tagttcttta ggcccgtagt ctgcaaatcc ttttatgatt
3421 ttctatcaaa caaaagagga aaatagacca gttgcaatcc aaacgagagt ctaatagaat
3481 gaggtcgaaa agtaaatcgc gcgggtttgt tactgataaa gcaggcaaga cctaaaatgt
3541 gtaaagggca aagtgtatac tttggcgtca ccccttacat attttaggtc ttttttttatt
3601 gtgcgtaact aacttgccat cttcaaacag gagggctgga agaagcagac cgctaacaca
3661 gtacataaaa aaggagacat gaacgatgaa catcaaaaag tttgcaaaac aagcaacagt
3721 attaaccttt actaccgcac tgctggcagg aggcgcaact caagcgtttg cgaaagaaac
3781 gaaccaaaag ccatataagg aaacatacgg catttcccat attacacgcc atgatatgct
3841 gcaaatccct gaacagcaaa aaaatgaaaa atatcaagtt cctgagttcg attcgtccac
3901 aattaaaaat atctcttctg caaaaggcct ggacgtttgg gacagctggc cattacaaaa
3961 cgctgacggc actgtcgcaa actatcacgg ctaccacatc gtctttgcat tagccggaga
4021 tcctaaaaat gcggatgaca catcgattta catgttctat caaaaagtcg gcgaaacttc
4081 tattgacagc tggaaaaacg ctggccgcgt ctttaaagac agcgacaaat tcgatgcaaa
4141 tgattctatc ctaaaagacc aaacacaaga atggtcaggt tcagccacat ttacatctga
4201 cggaaaaatc cgtttattct acactgattt ctccggtaaa cattacggca aacaaacact
4261 gacaactgca caagttaacg tatcagcatc agacagctct ttgaacatca acggtgtaga
4321 ggattataaa tcaatctttg acggtgacgg aaaaacgtat caaaatgtac agcagttcat
4381 cgatgaaggc aactacagct caggcgacaa ccatacgctg agagatcctc actacgtaga
4441 agataaaggc cacaaatact tagtatttga agcaaacact ggaactgaag atggctacca
4501 aggcgaagaa tctttattta acaaagcata ctatggcaaa agcacatcat tcttccgtca
4561 agaaagtcaa aaacttctgc aaagcgataa aaaacgcacg gctgagttag caaacggcgc
4621 tctcggtatg attgagctaa acgatgatta cacactgaaa aaagtgatga aaccgctgat
4681 tgcatctaac acagtaacag atgaaattga acgcgcgaac gtctttaaaa tgaacggcaa
4741 atggtatctg ttcactgact cccgcggatc aaaaatgacg attgacggca ttacgtctaa
4801 cgatatttac atgcttggtt atgtttctaa ttctttaact ggcccataca agccgctgaa
4861 caaaactggc cttgtgttaa aaatggatct tgatcctaac gatgtaacct ttacttactc
4921 acacttcgct gtacctcaag cgaaaggaaa caatgtcgtg attacaagct atatgacaaa
4981 cagaggattc tacgcagaca aacaatcaac gtttgcgccg agcttcctgc tgaacatcaa
5041 aggcaagaaa acatctgttg tcaaagacag catccttgaa caaggacaat taacagttaa
5101 caaataaaaa cgcaaaagaa aatgccgata acaaaaaaaa gcccgccgaa gcgggcttta
5161 ttaccaagcg aagcgccatt cgccattcag gctgcgcaac tgttgggaag ggcgatcggt
5221 gcgggcctct tcgctattac gccagctggc gaaaggggga tgtgctgcaa ggcgattaag
5281 ttgggtaacg ccagggtttt cccagtcacg acgttgtaaa acgacggcca gtccgtaata
5341 cgactcactt aaggccttga c
//
```
